# How AI-Adjudication Disrupts Law’s Ability to Facilitate Moral Perceptual Progress

**DOI:** 10.1007/s11023-026-09769-w

**Published:** 2026-03-03

**Authors:** Janna van Grunsven

**Affiliations:** Delft University of Technology, Delft, The Netherlands

**Keywords:** Law, Moral perception, Artificial Intelligence, AI-adjudication, Enactivism

## Abstract

Several philosophers of law have been drawing attention to the role of *moral perception* in modern legal practices. While perception-oriented approaches to law represent a minority view, I show that they offer a fruitful perspective on what is at stake with the emergence of Artificial Legal Intelligence (ALI). Specifically, I argue that facilitating *moral perceptual progress* is one of modern law’s vital aspirations, baked into its *origin story* as well as some of its *content* and *processes*. I argue that this aspiration threatens to be disrupted by ALI, which increasingly permeates the space of modern law. While my argument lands on a predominantly pessimistic assessment of ALI developments, I will conclude by speculating about potential positive ways in which ALI technologies may also support moral perceptual process.

## Introduction

Several philosophers of law have been drawing attention to the role of *moral perception* in modern legal practices (e.g. Amaya 2019; Delacroix 2022, 2025; Van Domselaar 2020, 2024). While perception-oriented approaches to law represent a minority view, I utilize and contribute to them here to show that they offer a distinctive and fruitful perspective on what is at stake with the emergence of Artificial Legal Intelligence (ALI). Across several dimensions, ALI developments disrupt modern law’s ability to facilitate *moral perceptual progress*, or so I argue. To make my case, I will begin by discussing what I mean by moral perception and moral perceptual progress. To do so, I build upon Iris Murdoch’s philosophy of moral perception, which I bolster with a theory of perception developed within *enactive embodied cognitive science* (enactivism). I will then discuss how perception, thus theorized, can be shaped, supported, and disrupted both by technologies as well as by modern law. Concerning modern law, I argue that facilitating moral perceptual progress is one of its vital aspirations, baked into its *origin story*, some of its *content*, and some of its *processes*. While some technologies have been conducive to law’s ability to work towards this aspiration, other technologies shape law in ways that undermine or erode this aspiration.^1^ Indeed, I argue that we have good reasons to believe that this applies to ALI technologies, which are increasingly permeating and disrupting the space of modern law. While my argument lands on a predominantly pessimistic assessment of ALI developments, I will conclude by speculating about positive ways in which the design and implementation of ALI technologies may also support moral perceptual process.

## What is Moral Perceptual Progress?

So, what do I mean by moral perceptual progress? To capture this, let me begin by drawing your attention to something that we typically take for granted but that could be considered quite remarkable: during our everyday interactions with other people, we typically perceive and respond to one another as minded beings who *matter*. In the ways in which we touch, look at, listen and attend to one another, we often express a recognition of human bodies as entities of a very particular kind: namely as bearers of psychologically rich agential and experiential lives that afford certain forms of responsiveness, attention, and care (Van Grunsven, 2022). We exhibit this perceptual responsiveness in the smallest of gestures and interactions: you might feel your child shivering from the cold and quickly bundle them up, acting on your perception of their experience of bodily discomfort. When, moments later, they express a readiness to play, you may habitually adjust your attunement to their embodied gestures, keeping them in view as the bearer of a rich ever-unfolding experiential life. This responsiveness is also operative in our more anonymous semi-automatized interactions with others. While we largely act on autopilot in our fleeting interactions with, say, a fellow passenger on the train, a cashier at a store, the physician at the doctor’s office, we still recognize them *as people*, whose embodied expressions invite (and foreclose) a range of more (or less) appropriate forms of engagement and interaction. In previous work, I have used the term moral perception to refer to this phenomenon (Van Grunsven, 2022; Shew & van Grunsven, 2024).

While moral perception, understood in this way, often unfolds in an effortless, nearly automatic manner, people’s moral visibility is not equally distributed. Some human lives are less readily seen as richly expressive, agential, and warranting ongoing responsiveness. Moral perceptual challenges are prevalent, for instance, in contexts where neurotypical people interact with autistic people.^2^ Autistic forms of embodied expressivity have a long, troubling history of being misperceived as pathological, devoid of meaning, psychologically thin, etc., which has resulted in a wide-spread dehumanization of autistic people, entrenched in fraught psychological research, therapeutic interventions, technological developments, laws, policy initiatives, interactions with law enforcement, etc. (Van Grunsven 2020; Botha, 2021). Similar concerns of moral misperception apply to the ways in which the bodies of many disabled people, aging people, Black people, women, and trans people, fail to be visible in shared interpersonal space in the right way. That is, they are less reliably visible as the bearers of psychologically rich experiential and agential lives that matter.

There is thus something paradoxical about our perceptual orientation towards the embodied lives of other people: on the one hand perceiving people *as* people is incredibly easy, it is something we do effortlessly, all the time in everyday lives. On the other hand, appropriately perceiving the bodily lives of others, and the moral demands that they impose upon is, incredibly hard, it is something we fail at all the time in everyday life (Van Grunsven, 2022). If it is true that we can fail and succeed in our perceptual orientation towards the bodily lives of others, then we can also make progress in this area. We can learn to see certain people *better*. For instance, a non-autistic parent can gradually come to acquire a more refined perceptual grasp of the meanings of the embodied expressions of their child, thereby learning to see their child in a richer way that more accurately recognizes her personhood.^3^ I thus understand moral perceptual *progress*, as the continual “piecemeal business” of bringing and keeping people in view as expressive selves with rich experiential lives of their own, and of resisting reductive reifying percepts of their lives (Murdoch,^ 1998^). In doing so, I am echoing a view articulated by Iris Murdoch.

In recent years, Murdoch’s view of moral perception has gained traction among some philosophers of law. Iris van Domselaar, for instance, proposes that Murdoch’s “vision-based moral philosophy” can help capture the ways in which “legal decision-making … deals with particulars, with concrete, unique (constellations of) facts and persons […] [which] require[s] a particular capacity on the part of the judge, the capability to adequately discern the particulars and respond accordingly” (2020). She proposes that this requires judges, like all “moral agents … to try to see *justly*,*”* which involves trying “to overcome prejudice, to avoid temptation, to control and curb imagination’ (Murdoch 1998, n27 332). Appeals to moral perception are also made by those who work in the space of law and law enforcement. For instance, in 2015, James Comey, then director of the Federal Bureau of Investigation, delivered a speech at Georgetown University, entitled “Hard Truths: Law Enforcement and Race,” in which he calls for an honest look at the deep-seated biases that continue to be operative in U.S. law-enforcement, detrimentally shaping how Black Americans are encountered and responded to in public space. One of the recurring thoughts in his speech is that “we must work … to really *see* each other.” Really seeing each other, Comey thus suggest, is an activity that can be improved upon through work.

The extent to which one finds this view of moral perceptual progress compelling, depends in part on one’s underlying philosophy of mind. For instance, it cannot gain a foothold if one accepts a Jerry Fodor-style computationalism that construes perception and cognition as distinct modules of the mind, where “one can conceptualize a module as a special-purpose computer with a proprietary database (Fodor, 1985, p. 3). According to this modular computational view, perception is seen as “informationally encapsulated,” which is to say that it is “isolated from much of the background knowledge to which cognitive processes have access**”** (Fodor, 1985, p. 1). Put plainly, perception provides descriptive information about things ‘out there’ in the world (there is a red apple on the counter) that is decoupled from (remains unaffected by and never constitutes) the kinds of cognitive processes vital for practicing law: judgment, deliberation, evaluation, adjudication, decision-making, etc. Though recent developments in cognitive science challenge this Fodorian picture, tenets of it continue to inform our sociotechnical imagination–including imaginings and pursuits in ALI, as I will suggest in a moment.

From a Fodorian-computational perspective, it is difficult to make sense of Murdoch’s idea of moral perception as an activity of responsiveness to the concrete, complex, psychologically rich lives of others. Murdoch’s view can be bolstered, however, via a different view of perception and cognition: enactivism (Van Grunsven J, 2015, 2022). Contra the computational picture, according to which human minds are best understood as computers, enactivism argues that mind is not analogous with an input-output machine but continuous with life. Living beings, including human beings, are porously embodied self-constituting entities, embedded in and dependent an environment that matters to them and to which they must adaptively respond in order to remain viable as precarious embodied self-constituting selves. The perceptual world that living beings, qua adaptive self-constitutive systems, are situated in is a world of *affordances*. *Affordances* is a term coined by ecological psychologist J.J. Gibson that refers to the value-laden perceivable possibilities for action available within a perceiver’s environment in virtue of their morphology, their sensorimotor skills, habits, needs and concerns. For instance, a hungry apple-eating mammal, whose morphology and sensorimotor skills enable moving and grasping, will typically perceive a red apple on the counter in value-laden terms, as “affording-to-be-grabbed-and-eaten.”

According to this enactive ecological perspective, human perceivers are situated in a vastly rich landscape of affordances, which we become habituated into through processes of enculturation (Rietveld & Kiverstein 2014). Echoing ideas central to Aristotelian virtue-ethics, Gibson termed this process the education of attention, which refers to a normative process by which we learn which affordances are salient in our communities and how these affordances are to be appropriately responded to: “this is what utensils afford,” “this is what kittens afford,” “this is what people afford.” The extent to which we jointly inhabit a shared world of affordances affects the ease with which we reliably perceive and respond to other people in the right way (Van Grunsven 2022, 2024). For instance, if we have both learned to perceive books as the sorts of things that afford concentrated reading, there is little perceptual ambiguity within me, as a perceiver, when I perceive you sitting quietly in a chair with your eyes moving across the pages folded open in your hands. The affordances of the book and the chair co-constitute my perception of you as affording to be left in peace with your intention to read, and I may immediately dampen my sounds and movements in response.

Still, even when we share an abundance of affordances and practical contexts of interaction, we are never safeguarded from moral perceptual failure: perceptual responsiveness to the rich expressive sense-making lives of others is “an endless task” (Murdoch, 1998, 317). This stems in part from the relational nature of human selves as beings who continually adapt to and develop within their sociomaterial environment through interactions with others (Van Grunsven s 2022). As such, people can never be pinned down as static quantifiable entities. People are the sorts of beings who require ongoing perceptual responsiveness and adjustment. Having a “fixed picture” of another is already to misperceive them. By contrast, to overcome such misperceptions and bring individuals in view as possessors of psychologically rich experiential lives that matter is to make moral perceptual progress.

## How Technology & Law Can Shape Moral Perception

If our moral visibility is facilitated by shared sociomaterial contexts of practical significance, then changes within those contexts, for instance changes brought about by new technologies, can also bring about changes in people’s moral visibility. In previous work I’ve focused on *Augmentative and Alternative Communication technologies* to illustrate this point. AAC-tech is used by non-speaking people, including non-speaking autistic people, to express themselves (Van Grunsven & Roeser 2022; Shew & Van Grunsven 2024). It is a form of technology that has contributed to a positive moral shift in how non-speaking autistic people are perceived: away from pathological bodies devoid of robust mindedness, towards a recognition of non-speaking people as possessors of psychologically rich meaningful lives that matter and that are worthy of interaction. Technologies can also render people morally invisible, for instance by reinforcing or perpetuating ableist views of who counts as a fully worthy embodied human self or by reducing people to fixed rigid stereotypes (Shew & Van Grunsven 2024) .

Much like technological artefacts, law can also steer moral perception. A crucial difference between technology and law lies in the latter’s self-conscious normativity. That is, technological artefacts typically do not wear their normativity on their sleeves, but often hide how their implicit scripts and affordances shape how we interact with and perceive other people.^4^ Law, by contrast, explicitly spells out, constrains, and legislates how people and their bodies ought (or ought not) to be treated and interacted with, which in turn shapes how people are perceived in a moral sense. As such, modern law, seems to be directed at facilitating moral perceptual change in a way that technological artefacts are not. In what follows, I will propose that modern law, at its best, aspires towards moral perceptual *progress* and that this is reflected on three different levels:

1. At the level of modern law’s origin story.
2. At the level of specific laws (the content of positive & case law).
3. At the level of judiciary processes—in terms of what judges and lawyers do (or ought to do), how defendants are seen (or ought to be seen), and what the court room means as a place of perceptual encounter.

While modern law’s origin story cannot be changed with the introduction of ALI technologies, levels 2 and 3 can. Below I explore how these changes may be to the detriment of law’s aspiration to function as a driver of moral perceptual progress.

## Moral Perceptual Progress as Connected to Modern Law’s Original Story & Its Content

The emergence of the rule of law is inextricably bound up with a moral shift in attitudes around how human persons ought to be perceived: as individuals with intrinsic moral standing. This shift in the conception of the individual person is not just a product of abstract rational Enlightenment theorizing. As Jay Berstein (2015) compellingly argues, it is wedded to a visceral normative perceptual change in the 1800s with respect to how people believed human bodies ought to be seen and treated by the state.^5^ Bernstein argues that, where initially interrogational and punitive torture of the individual, authorized by the state, was deemed acceptable, with grand public spectacles of tortured bodies serving a community-affirming role, the “moral force and urgency of the rule of law [was] tied to its being the counterimage to the sovereign law of torture [of the human body], such that every abrogation of the rule of law is experienced, implicitly or explicitly, as a threat to the very idea of human worth it inscribes.” (Bernstein, 2015, p. 74). If Bernstein is right, then the rule of law emerges as a project that not only aims to regulate society and human interaction, but that also aims to enact a society in which people are recognized in a particular way; as beings whose bodily existence is bound up with their moral standing, demanding legally enshrined practices that reflect and respect that standing.

To be sure, this moral perceptual reconfiguration of human embodiment was not, and has not been, extended equally to everyone. As I already sketched in the previous section, it continues to take ongoing effort to see all human bodies as the bearers of richly expressive sense-making lives that afford interactions appropriately reflective of their personhood. It would be naïve to presume that modern law has only played a progressive emancipatory role in facilitating such efforts. As anti-essentialist feminist philosophers of law have convincingly argued, the exclusion of marginalized groups and individuals is arguably built into modern law by design, in virtue of its universalist tenet. In Angela P. Harris’s words: “legal theory … has been entranced for too long and too great an extent by the voice of ‘We the People,’ … we need to subvert it with narratives, and stories, and accounts of the particular, the different, and the hitherto silenced.” (1990, 615). This silencing, it seems to me, has stemmed in part from the shaping effects that laws can have on how we perceive the lives of others. Various examples from positive law and case law reveal law’s orientation towards human bodies, texturing how these bodies are perceived, in ways that can be morally progressive but also dehumanizing. For the better, and for the worse, laws legislate:

1. Whose embodied lives matter (e.g. The Civil Rights Act of 1964; Violence Against Women Act; or, as a dark counterpart, the Nuremberg Laws).
2. What aspects of our bodily lives are seen as salient and where bodily ownership is to be located (e.g. Roe v. Wade and Dobbs v. Jackons).
3. Who has access to, and gets to be morally visible in, shared public space (the Americans with Disabilities Act (ADA), and, as its dark counterpart, ongoing efforts by the Trump administration to curtail LGBTQ rights and effectively render trans people invisible by disenabling full participation in public life).

I suggest that, in the best version of itself–a version that aligns with modern law’s own origin story as the rejection of state-sanctioned violations of human embodied moral standing–modern law aspires to facilitate moral perceptual progress and that it does so in part through some of its content (positive and case law).

## Moral Perceptual Responsiveness as Connected to Modern Law’s Processes

Moral perception also informs adjudicatory processes, by informing equitable discretion and directing judges’ responsiveness to defendants as concrete individuals with rich experiential lives who cannot be fully fixed or pinned down. In the words of Van Domselaar:

“the vision of the judge plays a key role in coming to grips with the moral nature of legal decision-making. … a judge, like any other human being, does not see a value-neutral world when confronted with a legal case, but rather faces an evaluative landscape that ‘invites’ him to respond” (Van Domselaar, 2020).

To be sure, the fact that a person’s evaluative perceptual perspective onto the world affects their discretionary adjudication has its share of problems. As Van Domselaar acknowledges, a perception-based view of adjudication might lack “the critical bite needed to prevent all kinds of *social* bias such as racism and sexism from influencing the judge’s perception” (2020). An enactive affordance-based approach to (moral) perception confirms the legitimacy of such worries: if perception is tied to our bodily situatedness, to our needs and concerns as embodied perceivers and to the sociomaterial environments and practices we inhabit, perception is always susceptible to bias and thwarted delusional ways of perceiving realities and other people.^6^ An example serves as a case in point: the ADA requires that employers provide disabled people with access to the labor market through the provision of “reasonable adjustments.” As such, the ADA can play a vital role in how disabled people are seen, both by disrupting a pernicious narrative surrounding disabled bodies as ‘unproductive and non-agential’ and by creating meaningful interaction-spaces within which non-disabled and disabled people perceive and interact with one another. However, in the ten years that followed the ADA’s implementation, 92% of court cases settled in favor of employers at the expense of disability access (Diller, 2000). This, in turn, creates precedent in case law that works against the kind of moral visibility and equal treatment that the ADA precisely aims to ensure. Similarly, despite the civil rights legislation in the 60s, the US have continued to grapple with rampant racism in processes of adjudication, both within the courts as well as in other governmental agencies in which discretionary judging is permitted, e.g.in law enforcement.^7^ In light of these concerns, you might conclude that, after Black people and disabled people have sought to claim their moral visibility as people with psychologically rich and meaningful lives that matter in public space, through sit-ins and events such as the Capital Crawl, and after this claim to moral visibility was recognized and encoded into positive law, it is precisely the value-laden perceptual orientation of judges, juries, and members of law enforcement, and the room for discretion that they enjoy, that can undermine important progress made in the domain of moral perceptual progress (Mayson, 2018). Below, I argue that this conclusion, though not exactly wrong, also shouldn’t be drawn too readily. This line of reasoning, which one-sidedly conceives of perception as inevitably a source of error and bias, feeds into a false narrative that can be co-opted by ALI tech-proponents in undesirable ways. 8

## Codification and Automation as Responses to Concerns of Perceptual Affective Bias

In the face of the above sketched problems, efforts to codify legal processes so as to minimalize or altogether eliminate discretionary decision-making, may have prima facie appeal. Whereas human judges may be perceptually and affectively biased, codified justice is recalcitrant to such arbitrariness and to the fluctuations within and between judges. This idea motivated some liberal legislators in the US to endorse the 1984 Sentencing Reform Act, which essentially stripped federal judges of their discretionary activities. It redefined judging from a complex case-by-case evaluative activity to one of mechanistic rule/decision-tree application, “yield[ing] a quantitative measure of justice more easily generated by a computer than by a human being.” (Stith & Cabranes, 1998, p. 169). As Andrea Roth puts it, the decision-making grid that resulted from this act “was so mechanistic … that federal officials created a ‘non-intellent computer program to execute them, referred to as the Applied Sentencing System (ASSYST),” which “arguably […] reduc[ed] judges to ‘sentencing machines’” (2015, 18).

The idea that computers and other machines could (or should) replace judges (or crucial parts of judiciary processes) finds a natural home in the earlier-discussed computational view of the human mind.^9^ In the 1980s, when, to speak with Fodor, computationalism was considered ‘the only game in town,’ the *General Robotics Corporation* spoke of its ambition to:

“replace the warm, living, human juries, with a cold, dead, robot jury so that citizens may have a plain and speedy adjudication or arbitration of their disputes. … We want to quantify what has been an emotional, prejudicial process and make it objective.” (Cited in Roth, 2015, 38–9).

Note how this way of framing adjudication presupposes the possibility and desirability of a computational modular adjudicatory process, in which rational decision-making both can and should be separated from the distorting effects of the affective dimensions of our cognitive lives. The above voiced aspiration of General Robotics Corporation looks highly questionable from an enactive embodied cognitive science standpoint, which maintains that cognition, as the ability to make sense of the world, is reserved for living breathing systems who are fundamentally affected by the world and who irreducibly occupy a perspective of concern, without which nothing would be perceived as salient in the first place. General Robotics Corporation’s aspiration was also met with derision by many who practice law and who recognized that legal codification always needs discretion as a distinctively human context-sensitive evaluative capacity, capable of providing a “safety valve [or] ‘circuit breaker in the State’s machinery of justice,’” by constraining the over-inclusive generalizing nature of rules and laws (Roth, 2015, 32).

Van Domselaar proposes that concrete perceptual encounters with the other play a vital role in providing such constraint: “to be confronted with a citizen in their concreteness, can occasion a temporary bracketing of law’s abstract mediating concepts and to literally and metaphorically look a citizen in the eye” (2024, 35). Such encounters, in which one is perceptually confronted with the specifics of a person’s life, have been known to challenge and disrupt generalizing over-inclusive decision-making on the part of judges (See Van Domselaar, 2024, p. 38).^10^ Stated negatively, the absence of concrete encounters, increasingly the case as automation-processes take over whole swaths of legal procedures, has been known to be conducive to organized irresponsibility, with jurists’ becoming morally alienated from the suffering of citizens (Van Domselaar, 2024, 36 − 7). Still, codification-heavy efforts in judicial contexts continue to exert appeal. Mayson (2018), for instance, entertains that: “At least on paper […] algorithms have distinct advantages over subjective [evaluations] […]. They eliminate the variability, indeterminacy, and apparent randomness—indeed, the subjectivity—of human prediction that has long pervaded criminal justice. They bring uniformity, transparency, and accountability to the task” (2280).^11^ Codification-heavy efforts in judicial contexts will only gain in prominence with the proliferation of ALI, which automates a range of processes that have traditionally depended on complex forms of human judgment, grounded in a person’s perceptual attunement to the specific case at hand. What will it mean for these perceptual dimensions to be disrupted by automated AI-driven processes?

## AI in Today’s Legal Landscape: A (*Very*) Brief Sketch

To begin answering that question, I will here offer a very brief and very general sketch of AI in today’s legal landscape.^12^ I will do so by drawing upon the work of Mireille Hildebrandt, who warns that:

due to the rapid and radical integration of algorithmic decision-systems and other types of data-driven intelligence into the administration of justice [… we] should pay close attention to the consequences of transformative and disruptive changes of the ICT infrastructure of our shared life world. If the technological embodiment of modern law and its offspring, the Rule of Law, is changed, the law itself will change—potentially beyond recognition (2017, 597).

Adding to her worry, my focal point, as stated throughout, is specifically on the disruption of law as facilitating moral perceptual progress.

To see how, it is helpful to first get a general sense of the two different types of AI used in legal contexts that Hildebrandt distinguishes, namely code-driven and data-driven AI. Code-driven AI is bound by an fully deterministic “if this then that” logic, where rules and decisions are built into the input-output system in advance “and basically consists of simple or complex decision trees” (2018, 2). In the case of data-driven AI, or machine learning, “the code is informed by the data on which it has been trained instead of being informed by legal experts that have translated their insights into code” (2018, 3). Such data consists of “legal text” in the form of “relevant preceding case law, statues, treaties and doctrinal treatises or to predict the outcome of future case law” (ibid.). Data-driven AI, Hildebrandt notes, “contains a new type of discretion” (ibid.). This discretion is not the one exercised by skilled judges and lawyers well-versed in law, who are responsive in real time to the complex specifics of a particular case, but discretion exercised by engineers who make a set of (often proprietary) design choices when training an algorithm. The ground for those choices, and the ability to contest them, often remains out of reach, not only for the average citizen who is subjected to algorithmic judicial decision-making, but also for jurists using AI for adjudicatory purposes. As Hildebrandt notes, in principle, the combination of data-driven and code-driven AI enables an entirely autonomous adjudicatory process. Data-driven ALI can “simulat[e] and predic[t] legal decisions, and if combined with code-driven regulation it can actually make decisions based on such simulation” (2018, 6).

To be sure, the full automatization of adjudication is not the reality we currently live in. However, it seems reasonable to assume that we will see increasing differences in how much automation will be permitted within different legal systems across the globe. Within the EU, concerns about a fully automated ALI-driven legal system or a deep reliance on ALI seem prima facie mitigated by the recent AI-act. The act labels AI-systems as “high risk” when they are “intended to be used by a judicial authority or on its behalf to assist judicial authorities in researching and interpreting facts and the law and in applying the law to a concrete set of facts,” adding that “The use of AI tools can support the decision-making power of judges or judicial independence, but should not replace it: the final decision-making must remain a human-driven activity.”^13^ While such stipulations matter, they don’t exactly mitigate concerns about ALI problematically interfering with acts of judging, or so I will argue in the next section. More generally, the increasing proliferation of AI technologies in various domains of law (forensics; supported decision-making; crime prediction efforts) suggests that, even within the EU, we are dealing with profound shifts that may disrupt law’s ability to facilitate moral perceptual progress. Moreover, with the US House of Representatives approving a budget bill that includes a measure prohibiting individual states from regulating AI technology, it is of emphatic importance to anticipate more extreme scenarios of ALI’s disruptive effects on human values and capacities. I offer some of these anticipations below.

## ALI and the Disruption of Legal Spaces & Processes

In legal contexts, much of what used to occur in person now already happens in digital spaces, which are often partly and sometimes fully governed by algorithmic prediction and decision-making. Concrete encounters between judges, defendants, juries, and lawyers, in which case-specific saliences are teased out in real presence, are declining. And while, as I have discussed, such real-life face-to-face encounters are far from innocuous, capable of reflecting problematic power asymmetries and biases that can negatively interfere with fair judiciary processes, we should not be too quick to give them up. Concrete encounters with the other, we saw with Van Domselaar, might be vital in an increasingly digitized judicial system, in which not only judicial interaction spaces but also the activities of judges and lawyers are becoming entangled with AI-driven automated processes. Both code-driven and data-driven AI depend, for their functioning, on quantifiable data points, that are either subsumable under or generative of a rule. This focus on quantifiability threatens to reify people and the qualities that might matter about them in the judicial context, disregarding the more ineffable non-codifiable aspects of a person’s life. Traditionally, and despite its flaws in practice, the modern court room context has in principle recognized “the moral personhood of the defendant and the moral dimension of crime and punishment,” as judges grapple with what it means to appropriately respond to the specifics of the defendant and the case at hand (Stith & Cabranes, 1998, p. 78). Judicial processes that are increasingly driven by quantifiable data to determine guilt, threaten to lose this vital dimension of law as a practice that, in principle, aims to perceive defendants in their particularity, as possessors of full complicated lives with unique histories.^14^

In their article “Developing Artificially Intelligent Judges,” Richard M. Re & Alicia Solov-Niederman convincingly anticipate that market-driven ALI companies will likely cease upon the flaws and weaknesses of human discretion to market their products:

“P]roponents of AI adjudication—particularly the profit-motivated firms that develop the technology—will have an incentive to criticize traditional modes of human judging …. and to celebrate the mechanized alternatives linked to codified justice. And those motivated criticisms will often land, given that there are in fact many serious (and often ignored) deficiencies in human adjudicators. Examples include the many cognitive biases, self-interested behaviors, and prejudices that human judges are known to exhibit. Even if AI adjudication also seems flawed and problematic, its relative appeal could still prompt disillusionment as to traditional human judging.” (Re & Solow-Niederman, 2019, pp. 272–3).

Even if the idea of a full-fledged robot judge is far-fetched, we have reasons to critically anticipate scenarios in which significant aspects of adjudication are replaced or supported by algorithms. In fact, Roth warns that this is the case even when jurists remain involved in the adjudicatory process: the presence of some element of human control might induce a sense of what Roth calls “false humanization,” which refers to the misleading assumption that any level of human involvement in AI-permeated processes suffices as a safety valve against the worries of codified law: “The mere fact that a mechanical process involves a layer of human intervention does not mean that the human is exercising complex individualized judgment entirely independent of the machine. Human operators or audiences interacting with machines tend too often to ‘defer to the wisdom of algorithms’ developing what technology scholars call ‘automation complacency” (2015, 27 − 8).^15^ This reveals the shortcomings of the EU AI-act’s stipulation that “the final decision-making must remain a human-driven activity.” To invoke Chief Justice Roberts of the US Supreme Court: “My worry is not that machines will start thinking like us. I worry that we will start thinking like machines.”

One way to cash out the worry here, is by asking how future judges and lawyers will be educated in preparation for a rapidly changing technologically-mediated judicial landscape. Valuable educational time might have to be devoted to the inculcation of skills that enable jurists to understand the workings and pitfalls of legal artificial intelligence. In this context, which will require increasing levels of digital literacy, it may seem overly idealistic, detached from the real world, to invest educational time in the fostering of capacities conducive to moral perception. After all, the legal landscape might be shifting to a space where moral perception is decreasingly put into practice. Relatedly, we have good reasons to worry about the impacts of losing exemplary individuals willing to take on the responsibility of engaging in the messy business of appropriately perceiving the particulars of a given complex case and of attending to citizens in a manner that tries to be responsive to them as full-fledged individuals with rich complicated sense-making lives that cannot be quantified.

## The Disruption of Legal Content

In addition to a disruption of adjudicatory processes, affecting what judges and lawyers do, how they are educated, and how defendants are seen, there is also a concern that AI-adjudication might stifle the legal landscape at the level of *content* due to an algorithmic conservatism that projects past rulings into the future. This conservatism is different (in the sense that it is more rigid) from the kind of conservatism tied to legal *precedent*, which still leaves some room for human discretion in ways that make change possible. As the American Bar Association puts it: “Of course, courts often hear cases where following precedent may lead—in the view of the judges for the case—to unjust outcomes. In those cases, the judges may offer reasons or legal nuances to avoid following precedential decisions or to outright overturn prior rulings.” Moreover, as Re & Solow-Niederman explicate, human adjudication is organically marked by “value-updating,” both because individual judgers change and mature as people, affecting their evaluative moral perceptual sense, and because there is a fluctuation in human judgers on the Bench. In the extreme case of AI-adjudication fully replacing human judges, these natural processes of value-updating would cease to exist (Re & Solow-Niederman, 2019). Thus, pre-existing views about how people’s embodied lives are appropriately interacted with and legislated would be repeated into the future, with no development or maturing within individual human judges or fluctuations between human judges (including generational changes on the bench) giving new direction to a reified legal and perceptual landscape: “Algorithmic prediction produces a precise reflection of digital data. […] To predict the future under status quo conditions is simply to project history forward” (Mayson, 2018, 2224). Perhaps one could artificially program some form of value-updating into a fully automated ALI system, but there are real limitations to such an approach. As Re & Solow-Niederman ask:

Would human governments and programmers be able to agree on how to implement [value] updates to those kinds of events? Further, what might be lost in translation between the formal content of the desired legal updates and the programming language that implements these updates? […] embracing AI adjudication without incorporating some form of updating risks putting hard data above dynamism and thereby making the legal system too rigid. (2019, 269)

Even in the more plausible, less extreme, cases of AI merely supporting or partially replacing adjudication, we have reasons to be concerned, given the aforementioned problems concerning automation complacency and lack of transparency regarding data-driven algorithmic codification. Once human discretion is discredited and AI-adjudication is (wrongly) embraced for its efficiency and seeming objectivity, judges, lawyers and legislators might be less inclined to challenge the outcomes of algorithmic predictions and decision-making, or to invoke non-quantifiable saliences about a defendant, as this will have already been discredited for all of its presumed flaws. What is more, the emphasis on quantifiable data, encouraged by AI-adjudication, may also have a bearing on how positive law is drafted up, as new laws that emerge in an age of AI-adjudication may be articulated in ways that are amenable to AI’s specific strengths and capacities. As Hildebrandt puts it: “Lawyers must urgently face the mutations that are re-shaping the environment of the law, taking note of the fact that these mutations will soon re-shape law’s own articulation, as its alignment with the technologies of the word is on the verge of being disrupted in favor of a novel alignment with the technologies of machine learning and other types of computational intelligence” (2016, 600, similar worries are raised by Roth, 2015 & Re & Solow-Niederman, 2019).

Finally, these various disruptions of the legal system may disincentivize individuals and groups of citizens from engaging in activities that can change the legal landscape in ways that could foster moral perceptual progress. In Sect. 5, I entertained the worry that after Black people and disabled people in America sought to claim their moral visibility in shared social space, through public sit-ins and through the Capital Crawl, and after these claims to moral visibility were encoded into positive law, that it was the perceptual orientation of judges, juries, and members of law enforcement, and the room for discretion that they enjoy, that still undermines important progress made in law’s ability to facilitate moral perceptual progress. While these worries are real, what I have shown in this section is that AI-driven codified justice does nothing to mitigate them. After all, if the entire system is organized in the manner described above, what kind of legal change could a person reasonably expect to effectuate by publicly claiming their moral visibility?

In sum, it seems to me that we have reasons to worry that ALI disrupts:

1. The court room as a specific perceptual environment that aspires to be responsive to defendants and cases in their concreteness and moral complexity,
2. The activities and training of jurists, who are traditionally tasked with exercising complex ethical judgments grounded in a perceptual evaluation of the case, which involves their ability to be affected by the people and facts in front of them,
3. The ways in which laws are formulated, toward quantifiable content and away from ambiguous non-quantifiable value terms that capture, in part, the idea that law aspires to do justice to people as embodied sense-making beings who have moral worth.
4. The sense among (marginalized) people that their moral invisibility can still be claimed and rectified by making a claim on the law.

All of this, it seems to me, erodes one of modern law’s aspirations: to facilitate moral perceptual progress. Thus, I agree with Mireille Hildebrandt that “If the technological embodiment of modern law and its offspring, the Rule of Law, is changed, the law itself will change—potentially beyond recognition.” (2016, 597).

## Where Does This Leave Us? Some Concluding Remarks

As somewhat of a techno-pessimist, I must admit that my concerns about AI infiltrating the rule of law far outweigh my sense of optimism. That said, it seems naïve to insist on an irretrievable prior state. What is more, romanticizing a past and a human capacity that is itself fraught with biases, with examples of moral perceptual failure abound, is perhaps equally problematic. I will try, then, if something positive in terms of ALI-design requirements might be retrieved, beyond a negative critique. This is all very tentative, I should note.

For starters, I want to raise the idea that the paradox of moral perception that I started with might be of help. To recall, I set up this paradox by highlighted the often-effortless habitual way in which we bring and keep people in view as beings who matter—a capacity that cannot be accomplished by machines, in part because it involves the ability to be affected by another. By emphatically claiming the ability to be affected as vital to our ability to adequately perceive and understand the lives of others, we can help bring out specifically what human jurists bring to the table and what makes them vital to processes of adjudication in ways that cannot be replaced by algorithms. An enactive approach to cognition, which decidedly contrasts with a computational approach, can be of explanatory use here. It helps to shed a light of suspicion on tendencies to view AI-generated output as authoritative and final, as capable of providing us with the fullest grasp of the relevant facts.

Of course, what the paradox of moral perception was meant to also bring out is that our nature as perceivers, situated in a normative landscape of affordances, can (indeed, often does) also obscure others from view, particularly those who are marginalized from dominant practices surrounding shared affordances. And, as we saw, this dark side of moral perception affects processes aimed at equitable discretion as well. Perhaps data-driven AI, which excels in pattern recognition, could function as a debiasing sparring partner for judges by flagging known areas and sources of moral perceptual failure. By articulating one’s discretionary decisions and making them subjectable to algorithmic review, moments of moral perceptual failure could be pinpointed, opened up for debate (and perhaps looped into a system that helps to flag such potential moments of moral perceptual failure for others). A suggestion similar in spirit can be found in Mayson (2018), who argues that inequalities, biases, and disparities “will be harder to see and to redress” “in the absence of algorithmic methods,” adding that “[r]ejecting the precise mirror of algorithmic prediction in favor of subjective [evaluation]… does not solve the problem. It merely turns a blind eye” (2018, 2281). If we don’t want to turn a blind eye, the hard question of course is how we might be able to utilize the strength of AI in judicial contexts in a manner that mitigates the series of concerns that I have raised along the way.

To confront such worries, Hildebrandt has proposed the idea of “agonistic” ALI development, where “agonism means to turn enemies into adversaries, vouching for a decision-making process that takes into account the concerns of those who will be affected” (2018, 7). We pursue such agonism by seeking out diverging perspectives and dissent throughout all stages of ALI design. This, Hildebrandt maintains, “will better ground what machine learning experts call ‘the ground truth’. Inviting dissent will also demonstrate ‘equal concern and respect’, which I dare say is the core of both democracy and the rule of law” (Ibid.). In a similar spirit, Sylvie Delacroix has recently proposed that large language models might be able to play a role in encouraging a certain ‘spirit of enquiry’ in jurists (Delacroix, 2025). The idea here is to situate data-driven AI in adjudicatory contexts, not so much as technologies that help us to better predict and understand allegedly objective value-neutral facts about the world. But, rather, as technologies capable of affording an exploratory playful engagement with data that could contribute to a jurist’s ongoing education of attention, to reinvoke Gibson. Delacroix suggests that this could precisely facilitate “the development of moral perception” as a capacity that develops through conversation with others who may perceive different aspects of the same situation.” By adopting a design framework that reenvisions LLMs as “transitional conversational spaces,” algorithmic systems could be designed in ways that don’t “prioritize informational transmission or behavioral prediction,” but that “measure their efficacy through their capacity to support the intersubjective negotiation of meaning across diverse interpretive frameworks” (2025, 41). While my pessimist intuitions are hard to shed, it is surely worth exploring whether implementations such as these could enable ALI to support rather than erode one of modern law’s vital aspirations. That is, it is worth exploring whether they could support, rather than disrupt, law as a site capable of facilitating moral perceptual progress.

## Data Availability

Not applicable.

## Footnotes

1 One example here is that of written text, dispersed through the printing press, and the specific affordances they have introduced (Hildebrandt, 2015).

2 This is sometimes referred to as the Double Empathy Problem (Milton, 2012).

3 I also think this applies to our perceptual responsiveness to living expressive beings more generally, but my focus in this paper is on people, as the rule of law is anthropocentric in its orientation.

4 This is evidenced by the fact that it is relatively uncontroversial to consider technological artefacts as neutral tools that can be used for good or bad, depending on a particular agent’s intentions. While philosophers of technology heavily debate the ontological status of technological artefacts and their normativity (see for instance Joseph Pitt’s, 2013 critique of Langdon Winner’s seminal essay “Do Artifacts have Politics”), I do take it to be common, in everyday parlance, to characterize of technologies as value-neutral tools that can be used for good or bad, but that do not in themselves embody normativity (this is anecdotal, but I would say a majority of my engineering students’ unexamined view of technologies corresponds with this common view).

5 He is building upon Cesare Beccaria’s *on Crimes and Punishment*.

6 For Murdoch, vision, how we perceive reality, is closely connected with language, how we describe reality often using concepts that are normatively thick. Feminist philosophers of law of highlight that many of the thick concepts used in law reflect a patriarchal world view (e.g. concepts such as “reasonableness” and “equality”) (Francis & Smith 2024).

7 See also Leslie & Smith, who point out that “even if the goal of stated legal equality is achieved, law provides no protection unless it is enforced. Feminist legal critics have argued that laws and treaties that exist on paper frequently are ignored in fact when they apply to women and contradict local customs and beliefs” 2024.

8 This posture towards perception can be traced to Rene Descartes and other early modern epistemologists and philosophers of mind.

9 Though, as Andrea Roth explains, such ambitions have been around for much longer than that: “Over the past 150 years, ‘the importance of the human sense’ has given way to the ‘silent testimony of instruments,” p.7 purporting “to administer justice with machine-like fairness” (2015, 8).

10 See van Domselaar, 2024.

11 Mayson is simultaneously highly aware of the limitations of algorithmic decision-making and the potential towards bias.

12 I believe the brevity of my discussion here is warranted by the focus of my paper, which is to motivate the idea that, in our assessments of ALI and its disruptive effects on legal practices, the lens of moral perception is a fruitful albeit largely overlooked one. I offer more specific examples of these disruptions in the next section of the paper.

13
http://data.europa.eu/eli/reg/2024/1689/oj.

14 As Andrea Roth warns: “Although a society may have a preexisting commitment to human rights, premised on benevolent aims in its problem-solving attempts, it may inadvertently end up violating human rights once it starts down the path of simplification” (footnote 221, page 30).

15 See also Hildebrandt: “human intervention becomes somewhat illusionary, because those who decide often do not understand the ‘reasons’ for the proposed decision. This induces compliance with algorithms, as they are often presented as ‘outperforming’ human expertise.” (Hildebrandt, 2018, p. 2).

## References

[CR6] Amaya, A. (2019). Virtuous adjudication; or the relevance of judicial character to legal interpretation. *Statute Law Review*, *40*(1), 87–95. 10.1093/slr/hmy033

[CR7] Bernstein, J. M. (2015). *Torture and dignity*. University of Chicago Press.

[CR8] Botha, M. (2021). Academic, activist, or advocate? Angry, entangled, and emerging: A critical reflection on autism knowledge production. *Frontiers in Psychology*, *12*, 727542. 34650484 10.3389/fpsyg.2021.727542 PMC8506216

[CR9] Colombetti, G. (2014). *The feeling body: Affective science Meets the enactive Mind*. MIT Press.

[CR10] Delacroix, S. (2022). *Habitual ethics?* (p. 304). Bloomsbury Academic.

[CR11] Delacroix, S. (2025). Designing with uncertainty: LLM interfaces as transitional spaces for democratic revival. *Minds and Machines*, *35*(4), 41.

[CR12] Diller, M. (2000). Judicial backlash, the ADA, and the civil rights model. *Berkeley Journal of Employment and Labor Law*, *21*, 19.

[CR13] Fodor, J. (1985). Précis to the modularity of mind. *The Behavioral and Brain Sciences*, *8*, 1–42.

[CR17] Francis, L., & Smith, P. (2024). Feminist Philosophy of Law. In Edward N. Zalta & Uri Nodelman (Eds.), *The Stanford Encyclopedia of Philosophy (Summer 2024 Edition)*.

[CR14] Harris, A. P. (1990). Race and essentialism in feminist legal theory. *Stanford Law Review*, *42*(3), 581–616.

[CR15] Hildebrandt, M. (2015). Smart technologies and the end (s) of law: Novel entanglements of law and technology. *Smart technologies and the end (s) of law*. Edward Elgar Publishing.

[CR16] Hildebrandt, M. (2018). Algorithmic regulation and the rule of law. *Philosohical Transcactions of the Royal Society* 376(2128). 10.1098/rsta.2017.0355 30082301

[CR30] Hildebrandt, M. (2017). The Force of Law and the Force of Technology, in M. McGuire & T.Holt (Eds), *The Routledge Handbook for Technology, Crime, And Justice*, Routledge .

[CR18] Mayson, S. G. (2018). Bias in, bias out. *Yale Law Journal*, *128*, 2218.

[CR19] Milton, D. E. M. (2012). On the ontological status of autism: The ‘double empathy problem’. *Disability & Society*, *27*(6), 883–887.

[CR20] Murdoch, I. (1998) The Idea of Perfection, in Existentialists and Mystics,Writings on Philosophy and Literature. AllenLane/the Penguin Press 99 1).

[CR23] Pitt, J. C. (2013). Guns don’t kill, people kill; values in and/or around technologies. In *The moral status of technical artefacts* (pp. 89–101). Springer.

[CR21] Re, R. M., & Solow-Niederman, A. (2019). Developing artificially intelligent justice. *Stanford Technology Law Review*, *22*, 242.

[CR22] Rietveld, E., & Kiverstein, J. (2014). A rich landscape of affordances. *Ecological Psychology*, *26*(4), 325–352.

[CR24] Roth, A. (2015). Trial by machine. *Geo LJ*, *104*, 1245.

[CR4] Shew, A., van Grunsven, J. (2024). walking and talking, rocking and rolling: Moral visibility in contexts of technology development. Kennedy Institute of Ethics Journal, 34(2), 155-190.https://doi.org/10.1353/ken.2024.a958992 10.1353/ken.2024.a958992 10.1353/ken.2024.a958992 40351175

[CR25] Stith, K., & Cabranes, J. A. (1998). *Fear of judging: Sentencing guidelines in the federal courts*. University Chicago.

[CR26] Van Domselaar, I. (2020). All judges on the couch? On Iris Murdoch and legal decision-making. In A. Amaya, & M. del Mar (Eds.), Virtue, emotion and imagination in law and legal reasoning (pp. 77–98).

[CR29] Van Domselaar, I. (2024). *Recht, ethiek En de Schreew Van Filoktetes—Wat juristen burgers verschuldigd Zijn*. Den Haag.

[CR1] Van Grunsven, J. (2015). Bringing life in view: An enactive approach to moral perception (Doctoral dissertation, The New School). https://www.proquest.com/openview/f14dfd1239a52b3e09222ec7ed5cfb0c/1?pq-origsite=gscholar&cbl=18750 https://www.proquest.com/openview/f14dfd1239a52b3e09222ec7ed5cfb0c/1?pq-origsite=gscholarcbl=18750

[CR2] VanGrunsven, J. (2022). Enactivism and the paradox of moral perception. Topoi, 41(2), 287–298 https://doi.org/10.1007/s11097-017-9500-8. 10.1007/s11097-017-9500-8

[CR3] Van Grunsven, J. (2025). Disabled body‐minds in hostile environments: Disrupting an ableist cartesian sociotechnical imagination with enactive embodied cognition and critical disability studies. Topoi, 44(2), 505–515. https://doi.org/10.1007/s11245-024-10080-5 10.1007/s11245-024-10080-5 10.1007/s11245-024-10080-5 PMC12064617 40352810

